# Harnessing Extracellular microRNAs for Diagnostics and Therapeutics in Acute Systemic Inflammation

**DOI:** 10.3390/cells13060545

**Published:** 2024-03-19

**Authors:** Russell Hollis, Monowar Aziz, Asha Jacob, Ping Wang

**Affiliations:** 1 Center for Immunology and Inflammation, The Feinstein Institutes for Medical Research, Manhasset, NY 11030, USA; rhollis1@northwell.edu (R.H.); maziz1@northwell.edu (M.A.); avarghes@northwell.edu (A.J.); 2 Department of Surgery, Zucker School of Medicine, Hempstead, NY 11549, USA; 3 Department of Molecular Medicine, Zucker School of Medicine, Hempstead, NY 11549, USA

**Keywords:** miRNA, sepsis, acute inflammation, extracellular vesicles, circulating miRNA, DAMPs, miRNA mimics

## Abstract

Micro-ribonucleic acids (miRNAs) are small sequences of genetic materials that are primarily transcribed from the intronic regions of deoxyribonucleic acid (DNAs), and they are pivotal in regulating messenger RNA (mRNA) expression. miRNAs were first discovered to regulate mRNAs of the same cell in which they were transcribed. Recent studies have unveiled their ability to traverse cells, either encapsulated in vesicles or freely bound to proteins, influencing distant recipient cells. Activities of extracellular miRNAs have been observed during acute inflammation in clinically relevant pathologies, such as sepsis, shock, trauma, and ischemia/reperfusion (I/R) injuries. This review comprehensively explores the activity of miRNAs during acute inflammation as well as the mechanisms of their extracellular transport and activity. Evaluating the potential of extracellular miRNAs as diagnostic biomarkers and therapeutic targets in acute inflammation represents a critical aspect of this review. Finally, this review concludes with novel concepts of miRNA activity in the context of alleviating inflammation, delivering potential future directions to advance the field of miRNA therapeutics.

## 1. Introduction

Micro-ribonucleic acids (miRNAs) were first discovered by Lee et al. as well as Wightman and Ruvkun in 1993 in the form of the *C. elegans lin-4* locus [1,2]. They consist of approximately 22 base pairs and are mostly from intronic regions of deoxyribonucleic acid (DNA) [3]. During their biogenesis, miRNAs are transcribed by RNA polymerase II or III, processed by RNAse III enzymes, and transported to the cytoplasm to regulate mRNA expression as part of an RNA-silencing complex (RISC) [3,4,5,6,7]. In addition to regulating mRNA expression through these silencing complexes, miRNAs also play a role in the degradation of mRNAs, though the exact mechanisms of this process remain controversial [4,7]. While first discovered as nucleic acids that remain intracellular, miRNAs have recently been found to work extracellularly as well [8,9]. These extracellular miRNAs can influence the activity of distant or adjacent cells and interact with other molecules in the extracellular space [9,10,11]. Overall, miRNAs serve multiple functions both intracellularly and extracellularly under physiologic and pathologic conditions [12,13].

miRNAs regulate several aspects of the immune system and consequently play a key role in the manifestations of acute inflammation, including sepsis. Sepsis is an acute inflammatory process in which the body’s immune system mounts a disproportionate response to local or systemic infection, leading to deleterious outcomes, such as organ failure or even death [14,15]. Recent estimates indicate that up to 11 million people die annually from sepsis worldwide [16]. Other acute pathologies that trigger disproportionate inflammatory responses, such as trauma and ischemia/reperfusion (I/R) injuries, are also associated with high mortality rates and critical illness [17,18]. Due to the magnitude of harm caused by these inflammatory processes, researchers continue to investigate these life-threatening diseases. Over the last two decades, miRNAs have emerged as actors in acute inflammation [19].

Thus far, researchers have investigated the expression and activity of many species of extracellular miRNA during acute inflammation [11,12,20]. Through in vitro experiments, human blood samples, and animal models, miRNAs have demonstrated their role in immune function and consequently immune dysfunction [11,12]. Certain miRNAs can be released outside of their native cells during acute inflammation: they can be exported in exosomes, freely circulating in a protein-bound state, carried by lipoproteins, or delivered to an adjacent cell through gap junctions [9,21,22,23]. These methods can occur either as a means of cell–cell communication or because of cell death [9,21,24]. While many unknowns remain in the field of extracellular miRNAs, they have a clear impact on the body’s ability to generate an inflammatory response.

In this review, we evaluate the current state of extracellular miRNAs as therapeutic targets in acute inflammation. First, we provide an overview of miRNA activity during acute inflammation with a focus on the extracellular functions of miRNAs and their potential impact on end organ damage. Next, we discuss the proposed mechanisms through which miRNAs are released into the extracellular space. We then examine their potential as biomarkers for diagnosing and monitoring acute inflammatory processes, such as sepsis. Lastly, we analyze the development of therapies that use extracellular miRNA inhibitors and mimics aimed at attenuating acute inflammation and its consequential downstream effects.

## 2. miRNAs in Acute Inflammation

miRNAs regulate several aspects of the immune system, affecting the level of acute inflammation present in diseases such as sepsis [11,25,26]. Intracellular miRNAs work in their native cells to silence mRNAs and modify the post-transcriptional process, while extracellular miRNAs are transported through extracellular vesicles and other forms to execute cell–cell communication [12,27]. In both intracellular and extracellular forms, the activity of miRNAs can trigger downstream effects on immune cells during acute inflammation.

In macrophages, miRNAs can influence pro-inflammatory and anti-inflammatory activity [28]. For example, during lipopolysaccharide- (LPS) or bacteria-stimulated lung inflammation in mouse models, macrophages demonstrate lower levels of intracellular miR-223 and -142 [29]. An increased number of these miRNAs is found in micro-vesicles, implying their release by macrophages during sepsis [29]. The decreased levels of these miRNAs inside macrophages are associated with higher levels of inflammation, which is then reduced when intracellular levels are repleted by the introduction of exogenous vesicles containing miRNA mimics [29]. In addition, miRNAs can also induce polarization of macrophages to M1 and M2 phenotypes. When exposed to TNF-α, neutrophils can produce exosomes containing miRNAs, such as miR-30b-5p [30]. These miRNAs then enter macrophages and contribute to their polarization to the M1 phenotype, resulting in increased inflammation [30]. Bone mesenchymal stem cells also transport miRNA, such as miR-17, in extracellular vesicles to macrophages during sepsis. Their extracellular transfer of miRNA reduces inflammation, increasing the concentrations of the M2 macrophage phenotype and decreasing the production of cytokines [31]. This phenomenon was observed with in vitro and in vivo models of LPS-stimulated sepsis [31]. Macrophages appear to be key targets of miRNAs from neutrophils and mesenchymal stem cells during acute inflammation. Macrophages are also susceptible to extracellular miRNA signaling via the toll-like receptor 7 (TLR7) pathway, which can result in a cytokine storm and end organ injury in mouse models of trauma and sepsis [32,33]. 

Neutrophils exhibit other actions besides macrophage polarization through miRNA activity, both as the recipients of miRNA gene regulation and the exporters of miRNAs to other cell types. As recipients, neutrophils are targeted by exosomes from bone marrow mesenchymal stem cells (BMSCs) containing miR-127-5p, which targets CD64 to reduce neutrophil extracellular trap (NET) formation [34]. This activity reduces acute lung injury (ALI) in mice during LPS-triggered sepsis [34]. Conversely, as exporters, neutrophils transfer anti-inflammatory miR-223 through vesicles to mouse lung epithelial cells, reducing their expression of poly-adenosine diphosphate ribose polymerase 1 (PARP-1) [35]. PARP-1, responsible for DNA repair, replication, and transcription can mediate a proinflammatory response through nuclear factor κB (NF-κB) and activator protein-1 (AP-1) [35,36]. Thus, repression of the *PARP-1* gene reduces inflammation and permeability in pulmonary epithelial cells [35].

In addition to influencing the activity of neutrophils and macrophages, miRNAs also affect the function of T lymphocytes in both tumor development and sepsis [37,38,39]. Sepsis, for example, can negatively affect the immune function of T lymphocytes, as demonstrated by Möhnle et al., who studied blood samples of septic patients and healthy volunteers [39,40]. They found a correlation between T cell immune-paralysis and the increased expression of anti-inflammatory miRNAs (miR-143 and -223) as well as the decreased expression of proinflammatory miRNAs (miR-150) [39,40]. The differential expression of these miRNAs also correlated with increased sequential organ failure assessment (SOFA) scores, a validated measurement of critical illness and predictor of sepsis severity in humans [39,40,41]. The SOFA uses laboratory values and other clinical data from six organ systems (neurological, respiratory, cardiovascular, hepatic, renal, and hematologic) to assess the severity of critical illnesses, such as sepsis, in relation to organ dysfunction and the risk of mortality [42,43]. In addition to these miRNAs that work in the cytoplasm of their native cells to influence T lymphocyte function, there are other miRNAs that work extracellularly on neutrophils and macrophages. These miRNAs participate in cell–cell transfer through vesicles in response to acute inflammation.

Extracellular vesicle miRNAs (EVmiRNA) can exacerbate or attenuate ALI observed in sepsis. ALI, which can develop into acute respiratory distress syndrome (ARDS), is a significant cause of morbidity and mortality in sepsis [15,44]. Exosomes containing miRNAs and cytokines have been observed in the bronchiolar secretions of septic mice stimulated by LPS [31]. Some miRNAs, such as miR-146a-5p, can enhance lung inflammation and endothelial dysfunction, whereas other species of miRNA suppress ALI in mice [30,33,34,45,46]. In a previously mentioned study, Zhang et al. instilled micro-vesicles containing miR-223 and miR-142 mimics into the respiratory tracts of mice stimulated with LPS or *Klebsiella Pneumoniae*, and these miRNA mimics reduced acute inflammation of the lung [29]. Endogenously, neutrophils transfer miR-223 to lung epithelial cells, and endothelial progenitor cells transfer miR-126 to lung endothelial cells to reduce inflammation [35,47]. Thus, transfer of miRNA via extracellular vesicles by neutrophils and endothelial progenitor cells can be lung-protective during acute inflammation [35,47]. 

As evidenced by their role in immune cell function, miRNAs can affect the course of acute inflammation, with some miRNAs acting intracellularly and others acting extracellularly. In the extracellular space, miRNAs can transport to nearby or distant cells, interact with cell surface receptors, or even bind to damage-associated molecular patterns (DAMPs), such as miR 130b-3p, which increases in expression during sepsis and binds to eCIRP [10]. During acute inflammation, some miRNAs increase in expression, and others decrease [24]. When comparing the blood of septic patients and healthy individuals, 77 types of miRNAs are downregulated, and 103 types of miRNAs are upregulated during sepsis [48]. Notably, more than one fifth of these miRNAs are extracellular in exosomes, serum, or both [48]. Many of the extracellular miRNAs are scarce or not detectable in intracellular compartments [48]. This finding seems to challenge the hypothesis made by Turchinovich et al. that exosome-free extracellular miRNAs are primarily the byproducts of cell lysis and death [9,48]. Through their differential expression, extracellular miRNAs demonstrate their activity in acute inflammation, sepsis, and its downstream effects pertaining to end organ failure, such as ALI and shock [47]. Given the compelling evidence of miRNA’s influence on acute inflammation, understanding the mechanisms behind extracellular miRNA transportation and gene regulation becomes paramount to detecting and treating diseases like sepsis and ALI.

## 3. Mechanisms of Extracellular miRNA Actions

**Figure 1:**
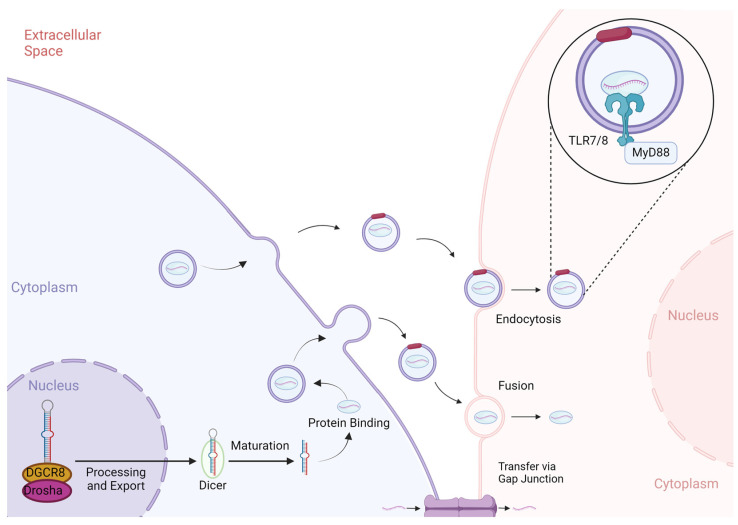
Extracellular miRNA can be assembled through various mechanisms. miRNAs can be transported to distant or adjacent cells through endocytosis, fusion, and via gap junctions. Therapeutics have targeted these mechanisms, such as the interaction between miRNAs and TLR7 or TLR8. DGCR8, DiGeorge Syndrome critical region 8; MyD88, Myeloid differentiation primary response 88; TLR, toll-like receptor. Created with Biorender.com.

miRNAs are first transcribed by RNA polymerase II or III into pri-miRNA duplexes [3,4,5,6,7]. Pri-miRNAs are then processed by RNAse III enzymes (Drosha and Dicer) while bound to DiGeorge Syndrome Critical Region 8 (DGCR8) and exported to the cytoplasm, at which point they become mature miRNA [4,5,6,7]. Mature miRNAs bind with Argonaute (AGO) proteins to form RISCs [4,5,6,7]. These complexes enable complementary binding to untranslated regions of mRNAs to execute posttranscriptional modification and repression of translation [25]. To a lesser extent, miRNAs can also silence mRNAs through binding to coding regions, or they can increase expression through binding of promoter sequences [5]. The entirety of this lifecycle can occur inside one cell, with the native cell (the cell that transcribed the miRNA) also acting as the recipient (the target of the miRNA’s activity) [4]. However, miRNAs can also be exported from the native cell to act on a different recipient cell (Figure 1) [9]. 

Extracellular miRNA activity predominantly exists in two forms: packaged within extracellular vesicles, such as exosomes, or exported in protein-bound states to the extracellular space, such as blood and other bodily fluids [21,48]. In addition, miRNAs can be chaperoned by lipoproteins into the extracellular space [23]. A fourth form that remains less well-described is the communication between two adjacent cells. Some cells, such as macrophages, can transfer miRNAs using gap junction pores formed by connexins [22]. Thus, miRNAs can affect cellular functions beyond the scope of the intracellular posttranscriptional modifications, which they were originally discovered to perform three decades ago [1]. This expanded understanding of miRNAs has helped reveal their role in acute inflammation, providing new opportunities to explore treatments of immune dysregulation, particularly in sepsis [10,49,50]. This section of the review will focus on the activity of extracellular miRNAs in vesicles and bound by proteins.

In humans, exosomes that transport miRNAs typically range in size between 30 and 150 nanometers (nm) [13,19]. Similarly, extracellular vesicles in septic mice are between 50 nm and 300 nm, and they are smaller on average than those in healthy control mice (mean size 157 nm vs. 191 nm) [49]. Initially thought of as byproducts of cell death, miRNA-containing extracellular vesicles have been investigated as possible avenues for cell–cell communication during homeostasis and stress [21]. Exosomes can interact with cells through surface receptors, fuse with the recipient cell membranes, or deliver their cargo through endocytosis by recipient cells [8,51]. 

To begin their life cycles, EVmiRNAs are sorted and chaperoned into exosomes [52]. For example, the Kirsten rat sarcoma viral oncogene homolog-mitogen-activated protein kinase kinase (KRAS-MEK) signaling pathway can play an inhibitory role in the sorting of Ago2-bound miRNAs into endosomes and then secretion into exosomes [53,54]. Synaptogamin-binding cytoplasmic RNA-interacting protein (SNYCRIP) can also sort miRNAs for exosomal packaging through binding with a human exoribonuclease (hEXO) motif on the miRNAs [55]. Lastly, Y-box protein 1 (YBX1) may also bind to certain miRNAs and designate them for exosomes [56]. In addition to these RNA-binding proteins, the relative concentrations of miRNA in the host cell can influence the sorting of these nucleic acids into vesicles [57]. Furthermore, certain sequences may determine the destination of miRNAs, such as GGAG and the aforementioned hEXO motif found in the miRNAs of T cell exosomes, which bind to heterogenous nuclear ribonucleoprotein A2B1 (hnRNPA2B1) [52,55,57]. While some miRNAs have been found exclusively in exosomes during in vitro and ex vivo experiments, some species of miRNA may have functions in both the native and the recipient cells [48,58].

After determining which miRNAs will be sorted into extracellular vesicles, there is a signal or trigger that drives the secretion and transit of the vesicle to its target [57,59]. In one example, exosomes containing miRNAs can be secreted via a ceramide pathway without the influence of endosomal sorting complexes required for transport (ESCRTs) [59]. While these pathways represent a few mechanisms through which cells can transport miRNAs into the extracellular space, a consensus on this process has not yet been reached [21].

Following secretion from the native cell, miRNAs can communicate with the recipient cell in several ways. One method of communication with the recipient cell is through interaction with cell surface receptors [8]. Extracellular vesicles that contain miRNAs in sepsis induce macrophages into the production of inflammatory cytokines via TLR pathways, specifically TLR7/MyD88 [49]. Additional methods include fusion of the vesicle with the recipient membrane and entrance into the cell through endocytosis [8]. For example, neutrophils have been shown to transfer anti-inflammatory miRNA-223 into macrophages, which decreases inflammation through downregulation of *PARP-1* rather than promoting bacterial phagocytosis and clearance by macrophages [35]. This phenomenon was demonstrated with in vitro transfection of miR-223 and in an in vivo experiment comparing wild-type mice and miR-223 deficient mice, who had similar bacterial clearance and increased PARP-1 [35].

Other than transport through extracellular vesicles, miRNA can also be exported into the extracellular space outside of a vesicle, stabilized by proteins that interfere with breakdown by RNases [60,61]. miRNAs can be packaged in exosomes and exported from the native cell into the extracellular space in an adenosine triphosphate (ATP)-dependent fashion [58]. For many extracellular miRNAs not in exosomes, binding with proteins is the stabilizing mechanism [9,60]. Protein-free, non-vesicular extracellular miRNAs are prone to degradation by nucleases [9,21,60]. Ago family proteins stabilize these miRNAs both in the cell and outside of the cell, protecting them from nucleases [9]. Another stabilizing protein outside of the Ago family, nucleophosmin 1 (NPM1), can also facilitate the transportation of miRNA, such as miR-122, into the cytoplasm and protect miRNAs from nucleases [58]. Regardless of the pairing, the main characteristic of the protein is an ability to prevent nucleases from degrading the miRNA.

While both extravesical and protein-bound miRNAs exist in the extracellular space, there remains some controversy regarding which population is more prominent and active in physiologic functions and responses to stress [60]. In some ex vivo experiments, the majority of extracellular miRNAs in blood cofractionated with proteins rather than vesicles [60]. Turchinovich et al. have reported that these miRNA-protein complexes are resistant to both nucleases and proteases [9]. In addition, researchers have explored the concept of extracellular miRNA as cell–cell communicators exported by living cells or products of cell death [21]. The presence of distinct sets of miRNAs in the extracellular milieu compared to the intracellular space, without any cell lysis/death, strongly suggests the active transport of miRNAs between functioning cells. This observation indicates that the extracellular movement of miRNAs does not necessarily rely on apoptosis or necrosis for its transport mechanism [58]. Through the discovery of these two forms of extracellular miRNA, further work has been performed with the aim of developing both diagnostics and therapeutics related to these small but influential nucleic acids.

## 4. Extracellular miRNAs as Diagnostic and Prognostic Markers

Often, a diagnosis of sepsis relies on clinical or laboratory criteria that mark disease progression, which can relegate treatment to a reactive rather than proactive approach [14,62]. Diagnosis through detection of miRNA levels in body fluids, such as blood, can improve efforts towards early detection and earlier treatment. Several miRNA species are differentially transcribed and released into the extracellular space during acute inflammation (Table 1) [24].

For instance, miR-15a, -27a, and -34a, which are involved in endothelial function and apoptosis, have demonstrated increased expression in the plasma of patients with sepsis, leading Goodwin and coauthors to postulate their use as early detection markers and prognostic indicators [24]. Using all three combined had the highest specificity and sensitivity when detecting sepsis in this study. In septic shock, miR-15a and -27a had decreased expression, while miR-34a had increased expression compared to its expression in non-shock sepsis [24]. These differences indicate the possibility of using miRNAs to both detect sepsis and to monitor disease progression. Another study used the expression of several miRNAs to create a model that would predict sepsis mortality, and they found that this model was more accurate than standard risk calculators, such as SOFA score, or individual biomarkers, such as procalcitonin [63]. One of these miRNAs, miR-122, has been shown to specifically correlate with liver injury in several studies [20]. These results indicate the possibility of measuring the changes of several miRNA species in sepsis to create a scoring system for predicting sepsis and sepsis severity. While these combined miRNA profiles can predict sepsis and sepsis severity, these and other miRNAs can provide information when measured individually as well.

miR-146 decreases during immune dysregulation in most studies [12]. Specifically, miR-146 has lower expression in septic inflammation compared to non-infectious systemic inflammatory response syndrome (SIRS) [64]. However, Xu et al. observed increased miR-146 concentrations in extracellular vesicles of septic mice [12,49].

**Table 1:** Selected extracellular miRNA used as diagnostics in sepsis.

miRNA	Expression (Increased or Decreased)	Outcome	Reference
miR-15a	Decreased	Shock, vascular permeability	Goodwin et al. (2015), Wang et al. (2012A) [24,63]
miR-16	Decreased	Increased mortality	Wang et al. (2012A) [63]
miR-27a	Decreased	Shock, vascular permeability	Goodwin et al. (2015) [24]
mir-34a	Increased	Shock, endothelial dysfunction	Goodwin et al. (2015) [24]
miR-122	Increased	Increased mortality	Wang et al. (2012A) [63]
miR-133	Increased	Presence of sepsis and severity	Benz et al. (2016), Tacke et al. (2014) [20,65]
miR-146	Decreased	Differentiates sepsis from SIRS	Formosa et al. (2022), Xu et al. (2018), Wang et al. (2013) [12,49,66]
miR-150	Decreased	Presence of sepsis	Vasilescu et al. (2009) [67]
miR-193	Increased	Increased mortality	Wang et al. (2012A) [63]
miR-223	Decreased	Differentiates sepsis from SIRS and severe sepsis from mild sepsis	Wang et al. (2012A) [63]
miR-297	Decreased	Increased mortality	Benz et al. (2016), Wang et al. (2012B) [20,68]
miR-483-5p	Increased	Differentiates mild and severe sepsis	Wang et al. (2012A) [63]
miR-574-5p	Increased	Increased mortality	Benz et al. (2016), Wang et al. (2012B) [20,68]

miR, microRNA; miRNA, microRNA; SIRS, systemic inflammatory response syndrome.

miR-150 has shown a relationship with the severity of inflammation, but its ability to detect sepsis is more controversial. miR-150 was negatively associated with TNF-α, IL-10, and IL-18 in a prospective study evaluating human plasma [67]. The ratio of miR-150 and IL-18 negatively correlated with the severity of sepsis [67]. On the other hand, Roderburg et al. found no significant difference in extracellular miR-150 concentrations between critically ill patients with and without sepsis, but there was an increase in morbidity and mortality that correlated with reduced concentrations of miR-150 [40]. These findings differed compared to research evaluating both intracellular and extracellular miRNA combined, which was able to use miR-150 to differentiate sepsis from sterile inflammation [69].

Many other miRNAs have shown a correlation with the incidence of sepsis, survival, and mortality [20,65,68]. Consequently, there are many different directions that future efforts can take when bringing miRNA as biomarkers to clinical practice. At the same time, there are obstacles that prevent the use of miRNA as diagnostic instruments currently. These limitations stem from the heterogeneity of results observed in the literature, both in direction and magnitude. As noted by Benz et al., miRNAs that demonstrate decreased concentrations in septic patients from one study may demonstrate increased concentrations in septic patients from a different study [20]. Some of these variations may have resulted from a lack of standardized methodologies [20]. For example, researchers may collect blood samples at different time points during a patient’s disease progression, and during analysis of the data, different references are used to normalize the change in expression of these miRNAs (e.g., SV40, RUN6B, and miR-192) [24,63,64,67]. Focusing on standardization of these techniques and then subsequently focusing on miRNAs that show consistent activity across standardized studies could lead to the development of diagnostic or prognostic tests that could improve the care of critically ill patients with different inflammatory pathologies [12,70].

## 5. Extracellular miRNA as Therapeutic Targets

As mentioned previously, extracellular miRNAs can interact with recipient cells through fusion or endocytosis of their vesicles, travelling intracellularly to influence posttranscriptional modification and translation. In addition, extracellular miRNAs free from vesicles can bind with cell surface receptors [8]. Extracellular miRNAs can bind to cell surface receptors to influence inflammation in a different way than is traditionally thought of miRNAs [71]. Lastly, they can interact with DAMPs rather than post-transcriptional regulators [49,72].

While some have argued that exosomes containing miRNAs do not maintain high enough concentrations to be physiologically relevant, others have found that the plasma contains higher concentrations of miRNAs than other RNAs [11]. In addition, there are differences when comparing the concentrations of miRNAs in the exosomes of septic individuals to those of healthy controls [24]. The differences in the concentrations of certain miRNAs have been shown to alter survival in murine models [73].

Some miRNAs can have proinflammatory effects, which can be inhibited to reduce harm during acute inflammation [33,49]. Extracellular vesicles containing these miRNAs interact with TLR7, resulting in cytokine production and complement activation, but this process can be attenuated with miRNA inhibitors to miR-34a-5p, -122-5p, and -146a-5p (Table 2) [49,74]. Exosomes with miRNAs have also demonstrated interaction with TLR8 in a similar manner, and the receptor may be a target for future miRNA inhibitor-based therapeutics [72].

Other miRNAs have beneficial immunomodulating activity [31]. Extracellular vesicles from bone mesenchymal stem cells can carry anti-inflammatory miRNAs, such as miR-17 [31]. This miRNA reduces LPS-induced inflammation through interaction with *bromodomain-containing protein 4 (BDR4)* in vitro in macrophages and in vivo in mice [31]. This interaction decreases *BDR4* expression, resulting in less cytokine release from macrophages and lower rates of apoptosis [31].

In a previously mentioned experimental model of LPS-induced endotoxemia, monoclonal antibody inhibitors of CD64 were shown to work synergistically with miR-127-5p to suppress NET formation and proinflammatory activity by neutrophils, which alleviated lung injury [34]. BMSC exported miR-127-5p within exosomes, which could be recapitulated as a therapeutic, along with use of the CD64 antibody [34].

Another miRNA, miR-142-5p, can travel inside extracellular vesicles to bind with and inhibit phosphatase and tensin homolog (PTEN) activity, thus promoting phosphoinositide 3-kinase/protein kinase B (PI3K/Akt) activity [50]. In a mouse model of cecal ligation and puncture (CLP)-induced sepsis, exosomal miR-93-5p inhibited *lysine-specific demethylase 6B (KDM6B)*, reducing TNF-α production and acute kidney injury (AKI) [75]. Furthermore, exosomes from endothelial progenitor cells have been observed to deliver miR-126 to lung endothelial cells, downregulating *sprouty-related EVH1 domain-containing 1 (SPRED1)* and promoting rapidly accelerated fibrosarcoma/extracellular signal-regulated kinase (RAF/ERK), which improved endothelial cell function and recovery [47]. This activity was not observed in miR-126 knockdown rats [47]. Lastly, keratinocyte growth factor (KGF), which can reduce alveolar inflammation, can be upregulated through the inhibition of *serum amyloid A-3 (SAA3)* expression [76]. miR-30b-3p, a potential therapeutic target, can inhibit *SAA3* and can be transferred by mesenchymal stem cells (MSCs) [46,76].

Extracellular miRNAs can be combined (e.g., loaded into the same vesicle) to have different effects from each individual miRNA on its own [77]. Pottash et al. combined miR-146a,-155,-223 to reduce inflammation in LPS-treated macrophages and mice [77]. While these miRNAs can have pro-inflammatory effects individually, there is an observed synergistic anti-inflammatory effect when all three are loaded into the same vesicle, evidenced by a reduction in IL-6 and other inflammatory markers [33,77,78,79]. However, other proinflammatory cytokines had variable expression. While this idea of combining miRNAs into one vesicle introduces more possibilities for using vesicular miRNAs to treat acute inflammation, the differential effects of these miRNAs under different conditions (combined or alone) demonstrate how complicated this approach can be and how much there is to learn about how miRNAs interact with each other.

**Table 2:** Extracellular miRNA-based therapeutics.

miRNA	Pathology	Mechanism of Action	Model	References
**Extracellular, Intra-Vesicular**				
miR-17	Sepsis	BMSC-EVs transport miRNAs to decrease expression of *BDR4*, reducing macrophage apoptosis and cytokine release	LPS intraperitoneal injection	Su et al. (2021) [31]
miR-30b-3p	Sepsis-induced ALI	MSC-EVs transport miRNAs to decrease expression of *SAA3*, increasing KGF and reducing cytokine expression and apoptosis of AECs	LPS intratracheal injection	Lee et al. (2009) [76]
miR-34a-5p miR-122 miR-146a	Sepsis	Inhibitors of miR-34a, miR-122, reduce signaling through TLR7/MyD88 pathway, reducing cytokine expression and neutrophil migration	CLP	Xu et al. (2018) [49]
miR-93-5p	Sepsis-induced AKI	EPC-secreted EVs silence *KDM6B* expression, reducing apoptosis and TNF-α via KDM6B/H3K27 pathway	CLP	He et al. (2020) [75]
miR-126	Sepsis-induced ALI	EPC-derived vesicles transport miRNAs to ECs and downregulate *SPRED1*, promoting RAF/ERK induced proliferation and angiogenesis	LPS intratracheal injection	Wu et al. (2018) [47]
miR-127-5p	Sepsis-induced ALI	BMSC vesicles transport miRNAs that bind to CD64, reducing its expression and NET formation	LPS intratracheal injection	Zheng et al. (2023) [34]
miR-142-5p	Sepsis	EVs with miRNA inhibit PTEN expression, activating the PI3K/AKT signaling pathway, reducing IL-6 and TNF-α production	LPS tail vein injection	Zhu et al. (2022) [50]
miR-146a	Sepsis	EVs with miRNAs induce inflammation through TLR7 signaling, which can be blocked by replacing all uridine with adenosine in miR-146a-5p	CLP	Huang et al. (2021), Wang et al. (2021), [33,79]
miR-146a, miR-155, miR-223	Sepsis	EVs with all three miRNAs induced variable expressions of cytokines	LPS intraperitoneal injection	Pottash et al. (2022) [77]
Extracellular, Extra-vesicular				
miR 27b	Sterile inflammation	miRNA mimic binds to eCIRP, inhibiting its interaction with TLR4	CIRP-induced inflammation (in vitro)	Gurien et al. (2020) [10]
miR 130-3p	Sepsis	miRNA mimic binds to eCIRP, inhibiting its interaction with TLR4	CLP	Gurien et al. (2020) [10]
miR 140	Sterile inflammation	miRNA mimic binds to eCIRP, inhibiting its interaction with TLR4	CIRP-induced inflammation (in vitro)	Gurien et al. (2020) [10]
PS-Ome miR 130	Sepsis, hepatic I/R, AKI	Modified/engineered miRNA mimic binds to eCIRP, inhibiting its interaction with TLR4	CLP, portal vein and hepatic artery occlusion for 60 min, bilateral renal artery and vein occlusion for 30 min	Borjas et al. (2023A), Borjas et al. (2023B), Vazquez et al. (2023) [80,81,82]
A_12_	Sepsis	Modified poly(A) tail binds to eCIRP, inhibiting its interaction with TLR4	CLP	Murao et al. (2023) [83]

AEC, alveolar epithelial cells; AKI, acute kidney injury; ALI, acute lung injury; *BDR4*, *bromodomain-containing protein 4*; BMSC-EV, bone marrow mesenchymal stem cell extracellular vesicles; CD64, cluster of differentiation 64; CIRP, cold-inducible ribonucleic acid-binding protein; CLP, cecal ligation and puncture; EC, endothelial cell; eCIRP, extracellular CIRP; EPC, epithelial progenitor cell; EKR, extracellular signal-regulated kinase; H3K27me4, Histone 3 with trimethylation at lysine 27; IL-6, Interleukin-6; *KDM6B*, lysine-specific demethylase 6B; KGF, keratinocyte growth factor; LPS, lipopolysaccharide; miR, micro-ribonucleic acid; MyD88, myeloid differentiation primary response 88; NETs, neutrophil extracellular traps; PI3K/AKT, Phosphoinositide 3-kinase/protein kinase B; poly(A), poly-adenosine; PS-Ome, phosphorothioate O-methyl; PTEN, phosphatase and tensin homolog; *SAA3*, *serum amyloid A-3*; RAF, rapidly accelerated fibrosarcoma; *SPRED1*, *sprouty-related EVH1 domain-containing 1*; TLR4, toll-like receptor 4; TLR7, toll-like receptor 7.

Along with delivery of miRNAs through extracellular vesicles and in protein-bound forms, additional delivery mechanisms for oligonucleotide therapeutics have emerged. Extracellular vesicles have the benefits of low toxicity and host immune response along with resistance to degradation in vivo, but they can be cost prohibitive and difficult to purify [73,84,85]. Viruses such as adenovirus- and retrovirus-based vectors can incorporate miRNA sequences and transfect them into hosts. However, they can also stimulate the immune system, can have mutagenic potential, and are limited by the therapeutic dose they can deliver [84,86,87]. In addition, scaffolds made from polymers, such as polyethylenimines (PEIs), have been investigated due to their potential efficiency in drug delivery, but toxicity is a concern for some of them [84,86,88]. Nanoparticles with gold, silicon, and other inorganic materials are also stable across different temperatures [84,89]. Lastly, lipid-based chaperones are commercially available, relatively inexpensive, and have been used in clinical trials, but they are not without risks [90,91,92]. Some authors have even postulated the ability to deliver miRNA therapeutics orally, given the stability that these delivery mechanisms provide [93]. Each method has advantages and disadvantages, but they all focus on delivery of the miRNA-based therapeutic into the target cell. Another strategy, which obviates the need to deliver miRNAs into the cell, is to harness the interaction between extracellular miRNAs and DAMPs.

**Figure 2:**
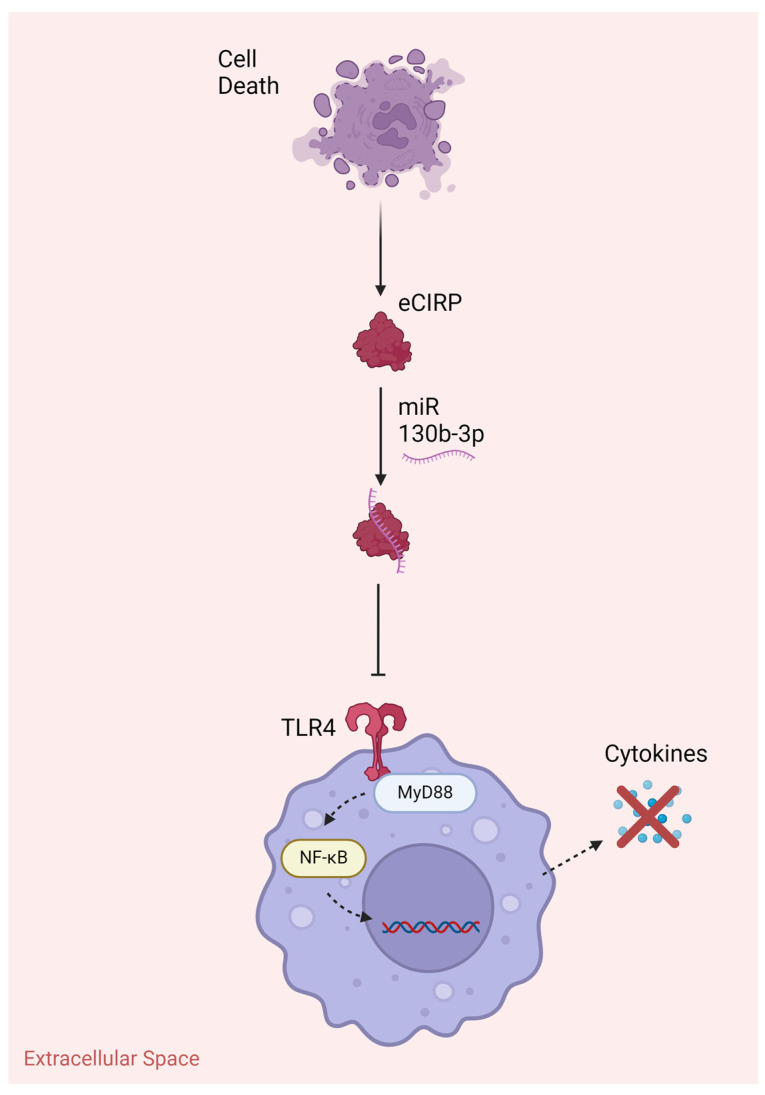
Extracellular miRNA mimics can be used as therapeutics that bind to and neutralize DAMPS, such as eCIRP, inhibiting the activation of inflammatory pathways that are caused by interaction with TLR4. eCIRP, extracellular cold-inducible RNA-binding protein; miR, microRNA; MyD88, Myeloid differentiation primary response 88; NF-kB, nuclear factor-kappa B; TLR, toll-like receptor. Created with Biorender.com.

In recent years, miRNAs have been evaluated in novel ways as therapeutics in acute inflammatory conditions, such as sepsis. Gurien et al. discovered the use of miRNA as a neutralizer of the proinflammatory effects of eCIRP, a known DAMP (Figure 2) [10]. Through the use of a mimic of miRNA 130b-3p, they found that miRNAs can bind to eCIRP and inhibit its ability to activate inflammatory cascades through the TLR4-mediated pathways [10]. Through in vitro models using macrophages with cotreatment of recombinant mouse CIRP (rmCIRP) and miR130b-3p, they observed a reduction in TNF-α and IL-6 [10]. This pattern continued in their in vivo CLP-induced sepsis model, with reduced organ injury markers, inflammatory markers, and lung injury after administration of miR130b-3p compared to administration of vehicle alone [10]. This study represented a novel use of miRNAs, known to release systemically after cell death, as molecules that can bind to DAMPs and reduce their ability to interact with cell surface receptors, such as TLR4, reducing the harmful effects of excessive inflammation.

Gurien et al. highlighted a significant challenge concerning the stability of miRNA mimics in the extracellular space. Unlike endogenous miRNAs, these mimics lack the protective shield of exosomes or the stabilization of protein-binding [10]. Consequently, various methods have emerged to enhance the stability of miRNAs and miRNA mimics in the extracellular space, especially miRNAs that are not protein-bound [80]. These modifications have the purpose of inhibiting breakdown by nucleases [80]. Known modifications include the substitution of oxygen on the phosphate backbone with sulfur, a borano group, or a methyl group, typically at the 5′- or 3′-ends of miRNA mimics. In addition, substitution of the 2′ oxygen of ribose with fluoride, a 2′O-methyl group, or a 2′O-methoxyl group can stabilize miRNA-based therapeutics [84,94,95]. Creating new bonds that reduce the flexibility and lock the structure of the nucleotide can prevent degradation [84]. Lastly, the addition of biotin, cholesterol, and other molecules can improve the half-life and delivery of miRNA mimics [84]. Each modification has positive and negative outcomes, such as the PS bond, which can protect miRNAs from nucleases but may result in a higher affinity to undesired protein targets and a lower affinity to the desired target [80,84,95]. Building upon the concerns of Gurien et al. regarding the stability of their miRNA-based therapeutic, researchers from the same laboratory endeavored to engineer a more resilient version of the miR 130b-3p mimic [80].

Borjas et al. modified miR 130b-3p to improve its stability in the extracellular space while maintaining its ability to inhibit eCIRP from binding to TLR4, attenuating harmful inflammatory responses during acute inflammation [80]. By adding a PS bond at the 5′- and 3′-ends and adding 2′O-methyl ribose groups to miR130b-3p, the authors were able to increase the half-life of miR130b-3p from minutes to several hours while maintaining a strong interaction with eCIRP (binding affinity similar to antigen and antibody reaction) at 50 nanomolar (nM), completely inhibiting the DAMP’s interaction with TLR4 [80]. With these pharmacokinetic properties, the modified miR130b-3p reduced cytokine expression and tissue injury while prolonging survival in a murine sepsis model [80].

This modified/engineered miRNA (PS-Ome miR130) was also effective at reducing maladaptive inflammation through inhibiting eCIRP’s activity in I/R models. For example, in hepatic I/R models of acute and sterile inflammation, Borjas et al. found that liver cell death, organ injury markers, and cytokine levels were reduced when mice were treated with PS-Ome miR130 after hepatic I/R [81]. In addition, Vazquez et al. demonstrated similar results in a renal I/R model used to recapitulate acute kidney injury [82]. The mice in this study also experienced improved survival after receiving PS-Ome miR130 [85]. This concept of using miRNA to inhibit the activity of pro-inflammatory molecules in the extracellular space provides a new avenue for the treatment of the body’s disproportionate immune response to sepsis, I/R, and trauma, which can reduce the high mortality rate of these diseases. At this time, research on miRNA-based therapeutics in acute inflammation has remained in the translational phase.

Clinical trials related to using miRNA in acute inflammation and shock states have thus far been limited to diagnostics (e.g., ClinicalTrials.gov identifiers: NCT03929159, NCT01459822, NCT05476029, NCY04815811). Investigators have attempted to ameliorate chronic inflammatory states and tumor progression with miRNA-based therapeutics [86]. For example, miR-34a administered through liposomal injection has been evaluated for the treatment of solid tumors, but progress has been slowed due to patient safety concerns [92,96]. As a drug class, miRNA therapeutics have not yet advanced beyond phase two trials, and miRNA-based therapeutics for acute inflammatory pathologies remain primarily preclinical [86,93,97].

## 6. Conclusions and Future Directions

Since the discovery of miRNA in 1993 and extracellular miRNA in the 2000s, novel discoveries continue to emerge and expand our understanding of the impact miRNA can have not only on intracellular activity but also on cell-cell communication and immune regulation [1,11,52]. With a greater understanding of miRNA’s function outside of the cell, researchers have evaluated several different species of miRNA as both diagnostic biomarkers of inflammation and targets for the treatment of diseases [10,40,77,79,80,81,82,98].

The first stage of developing miRNA therapeutics in acute inflammation is understanding the functions and locations of the miRNAs of interest, such as pro-inflammatory or anti-inflammatory and intracellular or extracellular (in exosomes or protein-bound and freely circulating) [9,71,99]. Knowing the function and location of the target miRNA could help determine the ideal delivery system, such as viral vectors, inorganic molecules, polymer-based scaffolds, lipid-based delivery systems, and exosomes. Advances have been made in viral and nonviral miRNA delivery systems used to introduce exogenous miRNA into the intracellular space [86]. Targeting circulating, extracellular miRNAs, however, could obviate the need to package therapeutics within exosomes or transfect them into the cell of interest with viral vectors, which both carry their own risks [80,86]. The challenge of targeting extracellular miRNAs remains a question of stability and the tradeoff between stabilizing the miRNA and altering its intrinsic properties [10,81].

The second stage involves optimizing miRNAs or miRNA mimics from a standpoint of safety, solubility, stability, and specificity to their targets. For example, a PS backbone and 2′-Ome modifications have improved the stability of the 130b-3p, but other modifications exist that could be explored [80,95]. Locked nucleic acids (linking the 2′-oxgen and 4′-carbon of ribose) could improve binding affinity [95]. Additional combinations including cholesterol-, biotin-, and amino-modified miRNAs could improve pharmacokinetics [84].

More pharmacokinetic studies are needed to determine the viability of modified miRNAs as drugs to ameliorate inflammation [80]. Some pharmacokinetic qualities, such as duration of action, bioavailability, and volume of distribution have been evaluated using modified oligonucleotides with sequences complementary to known miRNAs. The authors called these oligonucleotides antagomirs [100]. The properties of these antagomirs were evaluated in physiologic rather than pathologic conditions and on intracellular miRNAs rather than extracellular miRNAs, but modifications with PS bonds and 2′Ome groups showed great bioavailability and inhibition of miRNAs for up to 23 days [100].

With the emergence of artificial intelligence, a greater number of miRNA-based treatments can be explored through analysis of the entire human genome. Additional strategies can also be developed, such as the use of an engineered polyadenylated (poly(A)) tail to attenuate sepsis, which Murao et al. evaluated in a murine CLP model [83]. They discovered that a modified, stabilized poly(A) tail could also bind to DAMPs and inhibit their interaction with cell surface receptors, downregulating inflammatory cascades [83]. Further development of these miRNA-based therapeutics in terms of their pharmacokinetics, safety profiles, and delivery mechanisms could help advance this class of drugs beyond preclinical and early phase trials. In conclusion, this review provided a summary and analysis of miRNA in acute inflammation, the mechanisms of extracellular miRNAs, and the current approaches to therapies involving extracellular miRNAs. Looking ahead, there is optimism about leveraging our current knowledge of miRNA for the development of mimics, inhibitors, antagomirs, and other modified versions of these impactful oligonucleotides. These advancements aim to alleviate acute inflammation stemming from conditions like sepsis, ischemia/reperfusion injuries, and trauma.

## Data Availability

Not applicable.

## Abbreviations

AGO	Argonaute
AKI	Acute kidney injury
ALI	Acute lung injury
ARDS	Acute respiratory distress syndrome
AP-1	Activator protein-1
ATP	Adenosine Triphosphate
BDR4	Bromodomain-containing protein 4
BMSC	Bone marrow mesenchymal stem cell
CD64	Cluster differentiation 64
DAMP	Damage-associated molecular pattern
DGCR8	DiGeorge Syndrome Critical Region 8
DNA	Deoxyribonucleic acid
eCIRP	Extracellular cold-inducible RNA-binding protein
ERK	Extracellular signal-regulated kinase
ESCRT	Endosomal sorting complexes required for transport
EV	Extracellular vesicle
H3K27me4	Histone 3 with trimethylation at lysine 27
hEXO	Human exoribonuclease
hnRNPA2B1	Heterogenous nuclear ribonucleoprotein
I/R	Ischemia/reperfusion
IL	Interleukin
KDM6B	Lysine-specific demethylase 6B
KGF	Keratinocyte growth factor
KRAS-MEK	Kirsten rat sarcoma viral oncogene homolog-mitogen-activated protein kinase kinase
LPS	Lipopolysaccharide
mRNA	Messenger ribonucleic acid
miRNA	Micro-ribonucleic acid
MyD88	Myeloid differentiation primary response 88
MSC	Mesenchymal stem cell
NET	Neutrophil extracellular trap
NF-κB	Nuclear factor κB
nm	Nanometers
NPM1	Nucleophosmin 1
nsMase2	Neutral sphingomyelinase 2
PARP-1	Poly-adenosine diphosphate ribose polymerase 1
PEIs	Polyethylenimines
pre-miRNA	Precursor miRNA
pri-miRNA	Primary miRNA
PI3K/AKT	Phosphoinositide 3-kinase/protein kinase B
Poly(A)	Polyadenosine
PS-Ome	Phosphorothioate O-methyl
PTEN	Phosphatase and tensin homolog
RAF	Rapidly accelerated fibrosarcoma
RISC	RNA-silencing complex
rmCIRP	Recombinant CIRP
RNA	Ribonucleic acid
RNP	Ribonucleoprotein
SAA3	Serum amyloid A-3
SOFA	Sequential organ failure assessment
SPRED1	Sprouty-related EVH1 domain-containing 1
SV40	Simian virus 40
SYNCRIP	Synaptogamin-binding cytoplasmic RNA-interacting protein
TLR	Toll-like receptor
TNF-α	Tumor Necrosis Factor-Alpha
US	United States
YBX1	Y-box protein 1
